# Variational Method for Vibration Analysis of Elliptic Cylinders Reinforced with Functionally Graded Carbon Nanotubes

**DOI:** 10.3390/ma18010043

**Published:** 2024-12-26

**Authors:** Qingtao Gong, Tao Liu, Yao Teng, Binjie Ma, Xin Li

**Affiliations:** 1 Ulsan Ship and Ocean College, Ludong University, Yantai 264025, China; gongqt@ldu.edu.cn (Q.G.); tengyao@ldu.edu.cn (Y.T.); mabinjie@ldu.edu.cn (B.M.); lixin@ldu.edu.cn (X.L.); 2 Shandong Marine Aerospace Technolgy Inovation Center, Yantai 264025, China; 3 School of Civil Engineering, Central South University, Changsha 410075, China

**Keywords:** FG-CNTRC elliptic cylinders, modified variational approach, carbon nanotubes, least-squares weighted residual

## Abstract

This study introduces a novel analytical framework for investigating the vibration characteristics of functionally graded carbon nanotube-reinforced composite (FG-CNTRC) elliptical cylindrical shells under arbitrary boundary conditions. Unlike previous studies that focused on simplified geometries or specific boundary conditions, this work combines the least-squares weighted residual method (LSWRM) with an adapted variational principle, addressing high-order vibration errors and ensuring continuity across structural segments. The material properties are modeled using an extended rule of mixtures, capturing the effects of carbon nanotube volume fractions and distribution types on structural dynamics. Additionally, virtual boundary techniques are employed to generalize elastic boundary conditions, enabling the analysis of complex boundary-constrained structures. Numerical validation against existing methods confirms the high accuracy of the proposed framework. Furthermore, the influence of geometric parameters, material characteristics, and boundary stiffness on vibration behavior is comprehensively explored, offering a robust and versatile tool for designing advanced FG-CNTRC structures. This innovative approach provides significant insights into the optimization of nanoscale reinforced composites, making it a valuable reference for engineers and researchers in aerospace, marine, and construction industries.

## 1. Introduction

FG-CNTRC elliptic cylinders offer significant advantages due to their unique material and structural characteristics. The incorporation of carbon nanotubes (CNTs) as fillers endows these cylinders with exceptional mechanical properties, while their elliptical geometrical configuration provides superior structural performance. The distinctive combination of advanced nanomaterial reinforcement and elliptical morphology renders these composite cylinders highly promising for diverse engineering applications, including aviation, maritime engineering, and construction industries. Consequently, developing a comprehensive vibration analysis model that can accommodate various boundary conditions becomes crucial for understanding and optimizing the structural behavior of FG-CNTRC elliptical cylindrical shells.

In recent years, CNTs have emerged as a pivotal research focus in materials science, particularly within the domain of computational mechanics [1]. Lim [2,3] established a comprehensive modeling framework for CNT membranes, successfully investigating the physical properties of carbon nanotube materials at the microscopic scale. His research elucidated the dynamic characteristics of CNT materials on elementary structural elements such as beams and plates. CNTs have been extensively utilized as reinforcement phases in fabricating various metal [4] and non-metal matrix composite materials. Exemplary instances include CNT-reinforced copper matrix composites [5], CNT-reinforced nickel matrix composites [6], and CNT-reinforced ceramic matrix composites [7].

FG-CNTRCs are specialized materials characterized by carbon nanotubes distributed within the matrix according to specific functional gradient principles. Since its inception, numerous researchers have applied this concept across diverse engineering structural applications [8,9,10,11]. For instance, Thang [12], considering small-scale effects, investigated the vibration characteristics of FG-CNTRC nanoplates. The study explored the influence of non-local parameters on structural frequencies. Building upon his work, Shao [13] analyzed the stochastic vibration characteristics of FG-CNTRC rectangular plates. Lin [14] validated the applicability of the FSDT for the free vibration of FG-CNTRC beams and also compared it with higher-order shear theories (HSDT), obtaining consistent results. Hong [15] also established an FGM conical shell model utilizing the HSDT. While this theory demonstrates enhanced accuracy for thick structures, it simultaneously incurs increased computational costs. Rafiee et al. [16] analyzed the free vibration characteristics of FG-CNTRC beams with piezoelectric layers. To examine the large-amplitude vibration characteristics, the Euler-Bernoulli theory is applied. Ke et al. [17] studied the nonlinear vibration characteristics of FG-CNTRC beams based on Von Kármán and Timoshenko theories. In addition, he also [18] investigates the bending behavior of FG circle microplates based on the differential quadrature method (DQM). Using FSDT, Zhu et al. [19] employed the finite element method (FEM) to analyze the static and free vibration behaviors of CNTRC plates. Based on HSDT, Zhang et al. [20] analyzed the free vibration of FGCNTRC plates using the state-space Levy method. Malekzadeh [21] developed a modified DQM to analyze the free vibration behavior of quadrilateral laminated plates with CNTRC layers. Heydarpour [22] proposed a Mixed Navier-layerwise DQM to study the three-dimensional static behavior of FG-CNTRC plates. By using the perturbation method, Shen [23] primarily investigated the thermal buckling behavior of FG-CNTRC cylindrical shells, but focused only on several typical boundary conditions. Aragh et al. [24] applied an extended generalized DQM to analyze the vibration characteristics of FGCNTR cylindrical panels. Francesco et al. [25] used the DQM to examine the effects of various CNT types on the dynamic characteristics of hyperbolic shell structures. Heydarpour et al. [26] employed the FSDT and Hamilton’s principle to analyze the dynamic characteristics of FG-CNTRC rotating conical shells, focusing on the effects of CNT parameters, rotation speed, and rotation angle on the structure’s natural frequencies. Zhang et al. [27] explored the aero-thermo-elastic behavior of CNTRC cylindrical panels in supersonic flow. Additionally, the Ritz method is also a common approach for analyzing dynamic characteristics. For instance, Mirzaei et al. [28] and Hosseini [29] used this method to examine the natural frequencies of FG-CNTRC cylindrical panels. Using the Ritz method, Wang and his groups [30,31,32,33,34,35,36] analyzed the dynamic behavior of various CNTRC plate and shell structures under multiple typical boundary conditions. On the basis of the FSDT and Gram-Schmidt process, Kiani [37] conducted a study on the FG-CNTRC spherical panels under classical boundary conditions and presented the findings. Ansari [38] developed an analytical method to analyze the nonlinear postbuckling behavior of piezoelectric FG-CNTRC cylindrical shells, accounting for the combined influences of electro-thermal effects, axial compression, and lateral forces. Later, Ansari [39] and Torabi [40] investigated the buckling behaviors of axially compressed FG-CNTRC conical shells through variational formulation. 

Despite extensive research on the dynamic behaviors of FG-CNTRC structures, most studies are limited to simplified geometries such as beams, plates, and classical shell forms. These investigations often rely on specific boundary conditions, restricting their practical applicability to complex engineering problems. Furthermore, while advanced modeling techniques such as the DQM, Ritz method, and FEM have been employed, their computational efficiency and adaptability under general boundary constraints remain insufficient for more intricate geometries, such as elliptical cylindrical shells.

To address these limitations, this study presents a comprehensive and unified analytical framework for the free vibration analysis of FG-CNTRC elliptical cylindrical shells. By integrating the FSDT with an adapted variational principle and the LSWRM, this work ensures high accuracy and continuity across structural segments. Additionally, the introduction of virtual boundary techniques enables the modeling of generalized elastic boundary conditions, significantly enhancing the framework’s versatility. This novel approach not only bridges the existing gaps in literature but also provides valuable insights into optimizing the dynamic performance of FG-CNTRC elliptical shells, offering a robust tool for engineering applications in the aerospace, marine, and construction industries.

## 2. Theoretical Formulations

### 2.1. Geometric and Material Models

**Figure 1:**
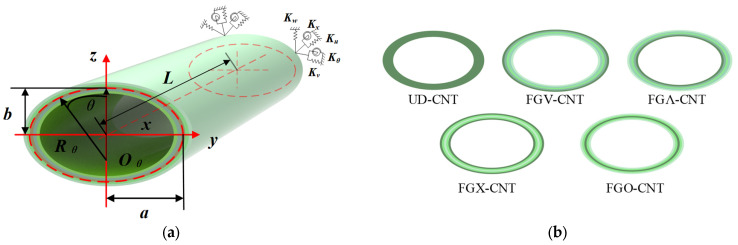
Geometric schematic of the elliptic cylindrical shell and cross-sectional view of FG-CNTRC material. (**a**) model schematic; (**b**) material distribution schematic.

Figure 1 depicts the geometric and material parameters of FG-CNTRC elliptic cylinders with arbitrary boundary conditions. In the orthogonal coordinate system, *x*, *θ*, and *z* represent the radial, circumferential, and thickness directions of the elliptical shell, respectively. The corresponding displacements are denoted by *u*, *v*, and *w*. The major and minor axes of the ellipse are represented by *a* and *b*, respectively. The mean radii *R_x_*(*θ*) and *R_θ_*(*θ*) of the cylindrical shell can be expressed as follows [41]:

(1) $$R_{\theta }(\theta )=\frac{a^{2}b^{2}}{\sqrt{{\left(a^{2}\sin^{2}\theta +b^{2}\cos^{2}\theta \right)}^{3}}}\;$$

(2) $$R_{x}(\theta )=0$$

This paper examines the types of CNT distributions, namely: UD, O, X, V, and Λ. Figure 1b shows the material distribution patterns for each type. Their functional expressions *V_CNT_* are as follows [30]:

(3) $$V_{CNT}\left(z\right)=\left[\begin{array}{cc} \left(\frac{2z}{h}+1\right)V_{tcnt} & \mathrm{FGV}-\mathrm{CNT}\\ \left(-\frac{2z}{h}+1\right)V_{tcnt} & \mathrm{FG\Lambda}-\mathrm{CNT}\\ 4\frac{\left|z\right|}{h}V_{tcnt} & \mathrm{FGX}-\mathrm{CNT}\\ 2\left(-2\frac{\left|z\right|}{h}+1\right)V_{tcnt} & \mathrm{FGO}-\mathrm{CNT}\\ V_{tcnt} & \mathrm{UD}-\mathrm{CNT} \end{array}\right]\left(-\frac{h}{2}\leq z\leq \frac{h}{2}\right)$$

in which,

(4) $$V_{tcnt}=\frac{w^{cnt}}{w^{cnt}+\frac{\rho^{cnt}}{\rho^{m}}-\left(\frac{\rho^{cnt}}{\rho^{m}}\right)*w^{cnt}}$$

The parameters that determine the effective Young’s modulus and shear modulus in the above equation are *ρ^m^*, *ρ^cnt^*, and *w^cnt^*, which represent the density of the matrix, the density of CNT, and the mass fraction of CNT [30]. It should be noted that in the following equations, the superscripts *cnt* and *m* represent the relevant parameters for CNT and the matrix, respectively.

(5) $$E_{11}\left(z\right)=\eta_{1}V_{CNT}\left(z\right)E_{11}^{cnt}+V_{m}\left(z\right)E^{m}$$

(6) $$\frac{\eta_{2}}{E_{22}\left(z\right)}=\frac{V_{CNT}\left(z\right)}{E_{22}^{cnt}}+\frac{V_{m}\left(z\right)}{E^{m}}$$

(7) $$\frac{\eta_{3}}{G_{12}\left(z\right)}=\frac{V_{CNT}\left(z\right)}{G_{12}^{cnt}}+\frac{V_{m}\left(z\right)}{G^{m}}$$

(8) $$\mu_{12}\left(z\right)=\frac{V_{CNT}\left(z\right)}{\mu_{12}^{cnt}}+\frac{V_{m}\left(z\right)}{\mu^{m}}$$

(9) $$\mu_{21}\left(z\right)=\frac{\mu_{12}\left(z\right)}{E_{11}\left(z\right)}E_{22}\left(z\right)$$

(10) $$\rho \left(z\right)=\frac{V_{CNT}\left(z\right)}{\rho^{cnt}}+\frac{V_{m}\left(z\right)}{\rho^{m}}$$

(11) $$V_{m}\left(z\right)=1-V_{CNT}\left(z\right)$$

Here, *E* and *G* represent Young’s modulus and shear modulus, while *η* and *μ* denote the efficiency parameter and Poisson’s ratio.

### 2.2. Domain Decomposition Model

**Figure 2:**
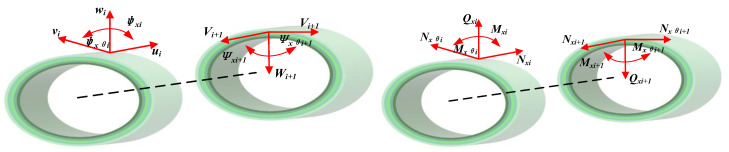
Displacement coordinate expressions of segmented cylindrical shell.

To accurately capture high-order vibration modes and responses, the method incorporates a multi-segment partitioning approach that divides the structure into segments for efficient computation. Accordingly, the FG-CNTRC elliptic cylinder is segmented into *N* parts along its length. When the piecewise structure is completed, the elliptic cylinder segment can be regarded as a substructure with a free boundary at both ends. Figure 2 illustrates the displacement relationship between adjacent subsectional structures. It is feasible to discretize the displacement field by using the admissible function because the boundary conditions of the elliptic cylinder segment should not be imposed at this time. The boundary conditions of the entire elliptic shell are simplified to include only the interfaces between adjacent segments and the actual geometric boundaries at both ends. A modified variational principle is applied to formulate the constrained system for FG-CNTRC elliptic cylinders. This approach works by finding the minimum of an adjusted variational function [42]:

(12) $$\prod =\sum_{i=1}^{N_{L}}\left(T_{i}-U_{i}+W_{i}\right)+\sum_{i,i+1}\prod_{\lambda k}+U_{BC}$$

where *i* denotes the segment number of the cylindrical shell, and *T_i_*, *U_i_*, and *W_i_* represent the kinetic energy, strain energy, and work performed by external forces, respectively. In the study of free vibrations, *W_i_* is zero. *T_i_* is defined as follows:

(13) $$T_{i}=\frac{1}{2}\int_{0}^{L}\int_{0}^{2\pi }\left\{\begin{array}{l} I_{0}\left[{\left({\dot{u}}_{i}\right)}^{2}+{\left({\dot{v}}_{i}\right)}^{2}+{\left({\dot{w}}_{i}\right)}^{2}\right]+\\ 2I_{1}\left({\dot{u}}_{i}{\dot{\psi }}_{x_{i}}+{\dot{v}}_{i}{\dot{\psi }}_{\theta_{i}}\right)+I_{2}\left[{\left({\dot{\psi }}_{x_{i}}\right)}^{2}+{\left({\dot{\psi }}_{\theta_{i}}\right)}^{2}\right] \end{array}\right\}R_{\theta }dxd\theta$$

where the (·) represents the time derivative. *I*_0_, *I*_1_ and *I*_2_ are the moment of intertia, defined as follows:

(14) $$\left(\begin{array}{ccc} I_{0} & I_{1} & I_{2} \end{array}\right)=\int_{-h/2}^{h/2}\rho \left(z\right)\left(1,z^{1},z^{2}\right)dz$$

The *U_i_* for the *ith* FG-CNTRC elliptic cylinders segment, is given by:

(15) $$U_{i}=\frac{1}{2}\int_{0}^{L}\int_{0}^{2\pi }\left(\Theta^{T}\cdot \tilde{N}+\Xi^{T}\cdot \tilde{M}+\Delta^{T}\tilde{Q}\right)R_{\theta }dxd\theta$$

where $\Theta^{T}={\left[\begin{array}{ccc} \varepsilon_{x}^{0} & \varepsilon_{\theta }^{0} & \varepsilon_{x\theta }^{0} \end{array}\right]}^{T}$ and $\Delta^{T}={\left[\begin{array}{cc} \gamma_{xz}^{0} & \gamma_{\theta z}^{0} \end{array}\right]}^{T}$ represents the strain matrix of the middle surface and $\Xi^{T}={\left[\begin{array}{ccc} \chi_{x} & \chi_{\theta } & \chi_{x\theta } \end{array}\right]}^{T}$ denotes curvature changes at any point of the FG-CNTRC elliptic cylinders segment. $\tilde{N}={\left[\begin{array}{ccc} N_{x} & N_{\theta } & N_{x\theta } \end{array}\right]}^{T}$ and $\tilde{Q}={\left[\begin{array}{cc} Q_{x} & Q_{\theta } \end{array}\right]}^{T}$ represents the force resultant vectors and $\tilde{M}={\left[\begin{array}{ccc} M_{x} & M_{\theta } & M_{x\theta } \end{array}\right]}^{T}$ denotes the moment resultant vectors.

Based on the FSDT, the membrane strains and curvature changes are defined as follows [41]:

(16) $$\varepsilon_{x}^{0}=\frac{\partial u_{i}}{\partial x},\;\varepsilon_{\theta }^{0}=\frac{1}{R_{\theta }}\left(\frac{\partial v_{i}}{\partial \theta }+w_{i}\right)$$

(17) $$\gamma_{x\theta }^{0}=\frac{\partial v_{i}}{\partial x}+\frac{1}{R_{\theta }}\frac{\partial u_{i}}{\partial \theta },\;\gamma_{xz}^{0}=\frac{\partial w_{i}}{\partial x}+\psi_{x_{i}},\;\gamma_{\theta z}^{0}=\frac{1}{R_{\theta }}\left(\frac{\partial w_{i}}{\partial \theta }-v_{i}\right)+\psi_{\theta_{i}}$$

(18) $$\chi_{x}=\frac{\partial \psi_{x_{i}}}{\partial x},\;\chi_{\theta }=\frac{1}{R_{\theta }}\frac{\partial \psi_{\theta_{i}}}{\partial \theta },\;\chi_{x\theta }=\frac{1}{R_{\theta }}\frac{\partial \psi_{x_{i}}}{\partial \theta }+\frac{\partial \psi_{\theta_{i}}}{\partial x}$$

This leads to the relationship between stress and strain as follows:

(19) $$\begin{array}{l} \left[\begin{array}{c} \tilde{N}\\ \tilde{M} \end{array}\right]=\left[\begin{array}{cc} A & B\\ B & D \end{array}\right]\left[\begin{array}{c} \Theta \\ \Xi \end{array}\right]\\ \tilde{Q}=C\Delta \end{array}$$

(20) $$\begin{array}{l} A=\left[\begin{array}{ccc} A_{11} & A_{12} & 0\\ A_{12} & A_{11} & 0\\ 0 & 0 & A_{66} \end{array}\right],B=\left[\begin{array}{ccc} B_{11} & B_{12} & 0\\ B_{12} & B_{11} & 0\\ 0 & 0 & B_{66} \end{array}\right]\\ D=\left[\begin{array}{ccc} D_{11} & D_{12} & 0\\ D_{12} & D_{11} & 0\\ 0 & 0 & D_{66} \end{array}\right],C=\left[\begin{array}{cc} \kappa A_{55} & 0\\ 0 & \kappa A_{44} \end{array}\right] \end{array}$$

The FSDT theory simplifies the transverse shear stress. After extensive research, *κ* = 5/6 is chosen as the equivalent shear coefficient. The expression for the stiffness coefficient (*A_ij_*, *B_ij_*, *D_ij_*) is:

(21) $$(A_{ij},B_{ij},D_{ij})=\int_{-h/2}^{h/2}Q_{ij}(z)(1,z,z^{2})dz$$

in which

(22) $$\begin{array}{l} Q_{11}\left(z\right)=\frac{E_{11}\left(z\right)}{1-\mu_{12}\mu_{21}},Q_{22}\left(z\right)=\frac{E_{22}\left(z\right)}{1-\mu_{12}\mu_{21}},Q_{12}\left(z\right)=\frac{\mu_{21}E_{11}\left(z\right)}{1-\mu_{12}\mu_{21}}\\ Q_{44}\left(z\right)=G_{23}\left(z\right),Q_{55}\left(z\right)=G_{13}\left(z\right),Q_{66}\left(z\right)=G_{12}\left(z\right) \end{array}$$

According to the existing literature research, it is not difficult to find that a large number of documents have elaborated the basic theory of modified variational functionals. The core of the proposed method is the construction of interface potentials as shown in Equation (12). To ensure numerical stability in higher-order calculations, the LSWRM is used, based on the MVP, to derive the interface potentials $\prod_{\lambda k}$, which are expressed as follows [43]:

(23) $$\begin{array}{l} \prod_{\lambda k}=\int_{l}\left(N_{x}\gamma_{u}+N_{x\theta }\gamma_{v}+Q_{x}\gamma_{w}+M_{x}\gamma_{x}+M_{x\theta }\gamma_{\theta }\right)dl\\ -\frac{1}{2}\int_{l}\left(\Gamma_{u}\gamma_{u}^{2}+\Gamma_{v}\gamma_{v}^{2}+\Gamma_{w}\gamma_{w}^{2}+\Gamma_{x}\gamma_{x}^{2}+\Gamma_{\theta }\gamma_{\theta }^{2}\right)dl \end{array}$$

In the formula, *γ* represents the continuity equations for adjacent segments. Their detailed forms are as follows: $\gamma_{u}=u_{i}-u_{i+1}$,$\gamma_{v}=v_{i}-v_{i+1}$,$\gamma_{w}=w_{i}-w_{i+1}$, $\gamma_{x}=\psi_{x_{i}}-\psi_{x_{i+1}}$ and $\gamma_{\theta }=\psi_{\theta_{i}}-\psi_{\theta_{i+1}}$. To maintain continuity at the interfaces, a weighting parameter Γ*_t_* (*t* = *u*, *v*, *w*, *x*, *θ*) is also introduced.

Most existing numerical methods are primarily restricted to handling classical boundary conditions. However, there are still some difficulties in solving general elastic boundary problems. Thus, the virtual boundary technology is introduced to obtain general elastic boundary conditions in this paper [44,45]:

(24) $$\begin{array}{l} U_{BC}=\frac{1}{2}\int_{l}\left\{k_{u,0}u_{1}^{2}+k_{v,0}v_{1}^{2}+k_{w,0}w_{1}^{2}+k_{x,0}\psi_{x_{1}}^{2}+k_{\theta ,0}\psi_{\theta_{1}}^{2}\right\}dl\\ +\frac{1}{2}\int_{l}\left\{k_{u,1}u_{N}^{2}+k_{v,1}v_{N}^{2}+k_{w,1}w_{N}^{2}+k_{x,1}\psi_{x_{N}}^{2}+k_{\theta ,1}\psi_{\theta_{N}}^{2}\right\}dl \end{array}$$

where $k_{u,0or1},k_{v,0or1},k_{w,0or1}$ represent the stiffness values for three types of linear springs, while $K_{x,0or1},K_{\theta ,0or1}$ correspond to the stiffness values for two types of rotational springs. By adjusting the values of the five springs, any boundary condition can be achieved.

### 2.3. Jacobi Orthogonal Polynomials

In this approach, the motion equations for FG-CNTRC elliptic cylinders are discretized using permissible displacement functions $\left(\begin{array}{ccccc} u_{i} & v_{i} & w_{i} & \psi_{x_{i}} & \psi_{\theta_{i}} \end{array}\right)$. The theoretical framework, as outlined earlier, is developed through a combination of the MVP and the LSWRM. Therefore, the requirement for a displacement allowable function for shell subdivision is greatly reduced. It only needs to satisfy the continuity, without considering the influence of the subdivision boundary. In addition, all segments can be represented by the same displacement admissible functions, which satisfy only linearly independent, complete, regular differentiable. This analysis employs harmonic functions to describe circumferential expansion. The displacement components of each FG-CNTRC elliptic cylinders segment can be written as follows [46]:

(25) $$\begin{array}{ll} u_{i}\left(x,\theta ,t\right) & =\sum_{m=0}^{M}\sum_{n=0}^{N}U_{m}P_{m}^{\left(\alpha ,\beta \right)}(x_{i})\left(\cos\left(n\theta \right)+\sin\left(n\theta \right)\right)e^{i\omega t}\\ & ={\bar{U}}_{m}U\left(x,\theta \right)e^{i\omega t} \end{array}$$

(26) $$\begin{array}{ll} v_{i}\left(x,\theta ,t\right) & =\sum_{m=0}^{M}\sum_{n=0}^{N}V_{m}P_{m}^{\left(\alpha ,\beta \right)}(x_{i})\left(\sin\left(n\theta \right)+\cos\left(n\theta \right)\right)e^{i\omega t}\\ & ={\bar{V}}_{m}V\left(x,\theta \right)e^{i\omega t} \end{array}$$

(27) $$\begin{array}{ll} w_{i}\left(x,\theta ,t\right) & =\sum_{m=0}^{M}\sum_{n=0}^{N}W_{m}P_{m}^{\left(\alpha ,\beta \right)}(x_{i})\left(\cos\left(n\theta \right)+\sin\left(n\theta \right)\right)e^{i\omega t}\\ & ={\bar{W}}_{m}W\left(x,\theta \right)e^{i\omega t} \end{array}$$

(28) $$\begin{array}{ll} \psi_{x_{i}}\left(x,\theta ,t\right) & =\sum_{m=0}^{M}\sum_{n=0}^{N}\Psi_{x_{m}}P_{m}^{\left(\alpha ,\beta \right)}(x_{i})\left(\cos\left(n\theta \right)+\sin\left(n\theta \right)\right)e^{i\omega t}\\ & ={\bar{\Psi }}_{x,m}\Psi_{x}\left(x,\theta \right)e^{i\omega t} \end{array}$$

(29) $$\begin{array}{ll} \psi_{\theta_{i}}\left(x,\theta ,t\right) & =\sum_{m=0}^{M}\sum_{n=0}^{N}\Psi_{\theta_{,m}}P_{m}^{\left(\alpha ,\beta \right)}(x_{i})\left(\sin\left(n\theta \right)+\cos\left(n\theta \right)\right)e^{i\omega t}\\ & ={\bar{\Psi }}_{\theta ,m}\Psi_{\theta }\left(x,\theta \right)e^{i\omega t} \end{array}$$

where ${\bar{U}}_{m}$, ${\bar{V}}_{m}$, ${\bar{W}}_{m}$, ${\bar{\Psi }}_{x,m}$ and ${\bar{\Psi }}_{\theta ,m}$ are the generalized coordinate vector. $U\left(x,\theta \right)$,$V\left(x,\theta \right)$, $W\left(x,\theta \right)$, $\Psi_{x}$ and $\Psi_{\theta }\left(x,\theta \right)$ represent the corresponding coefficients for the Jacobi expansion.

$U_{m}$, $V_{m}$, $W_{m}$, $\Psi_{x,m}$ and $\Psi_{\theta ,m}$ are the corresponding Jacobi expanded coefficients. $P_{m}^{\left(\alpha ,\beta \right)}(x)$ denotes the *m*-th order Jacobi polynomial along the length direction. In fact, Jacobi polynomials can be considered an extension of various orthogonal polynomials. By choosing different values for *α* and *β*, they can evolve into well-known polynomials like Legendre and Chebyshev.

By substituting Equations (13)–(24) into Equation (12) and performing the variational operation, the equation of motion for the structure is obtained as follows:

(30) $$-M\omega^{2}E+\left[K-{\bar{K}}_{\lambda }+{\bar{K}}_{\gamma }+{\hat{K}}_{B}\right]E=0$$

## 3. Numerical Results and Discussion

In this section, the established dynamic model is validated and analyzed. First, the convergence of the algorithm is examined. Following this, the free vibration characteristics of the elliptic cylinder are investigated. Finally, the effects of geometric and material parameters on the vibration behavior of FG-CNTRC elliptic cylinders are discussed. 

For the numerical examples that follow, unless stated otherwise, *a* = 2 m, *b* = 1 m, *L* = 3 m and *h* = 0.1 m are used as the geometric parameters of the elliptic cylinder, while *E^m^* = 2.5 GPa, *μ^m^* = 0.34, *ρ^m^* = 1150 kg/m^3^,$E_{11}^{cnt}=5646.6$ GPa, $E_{22}^{cnt}=7080$ GPa, $G_{12}^{cnt}=1944.5$ GPa, $\mu_{12}^{cnt}=0.175$ and *ρ^cnt^* = 1150 kg/m^3^ serve as the material parameters. Three types of equivalent CNT parameters are selected as: Type 1: *V_tcnt_* = 0.12, *η*_1_ = 0.137, *η*_2_ = 1.022, *η*_3_ = 0.715; Type 2: *V_tcnt_* = 0.17, *η*_1_ = 0.142, *η*_2_ = 1.626, *η*_3_ = 1.138; Type 3: *V_tcnt_* = 0.28, *η*_1_ = 0.141, *η*_2_ = 1.585, *η*_3_ = 1.109. Additionally, the dimensionless natural frequency is defined as: $\Omega =\omega L^{2}/h\sqrt{\rho^{m}/E^{m}}$. For simplicity, five types of boundaries are set: 

Free (F): $k_{u,0,1}=k_{v,0,1}=k_{w,0,1}=K_{x,0,1}=K_{\theta ,0,1}=0$; 

Shear Diaphragm Supported (SD) $k_{v,0,1}=k_{w,0,1}=10^{15},k_{u,0,1}=K_{x,0,1}=K_{\theta ,0,1}=0$; 

Simply Supported (S): $k_{u,0,1}=k_{v,0,1}=k_{w,0,1}=K_{\theta ,0,1}=10^{15},K_{x,0,1}=0$; 

Clamped (C): $k_{u,0,1}=k_{v,0,1}=k_{w,0,1}=K_{x,0,1}=K_{\theta ,0,1}=10^{15}$;

and Elastic (E): $k_{v,0,1}=k_{w,0,1}=k_{u,0,1}=K_{x,0,1}=K_{\theta ,0,1}\neq 0$.

### 3.1. Convergence of Current Method

The numerical analysis involves multiple computational parameters, including the number of segments (*N*), truncation order (*M*) of the Jacobi orthogonal polynomials, and the weighting parameter (*κ*). The convergence characteristics of these parameters have been thoroughly investigated by Qu et al. [47]. Following their established findings, this study adopts *N* = 4, *M* = 14, and *κ* = 10^14^, while the effects of boundary spring stiffness and Jacobi polynomial coefficients will be examined in detail in the subsequent sections.

**Figure 3:**
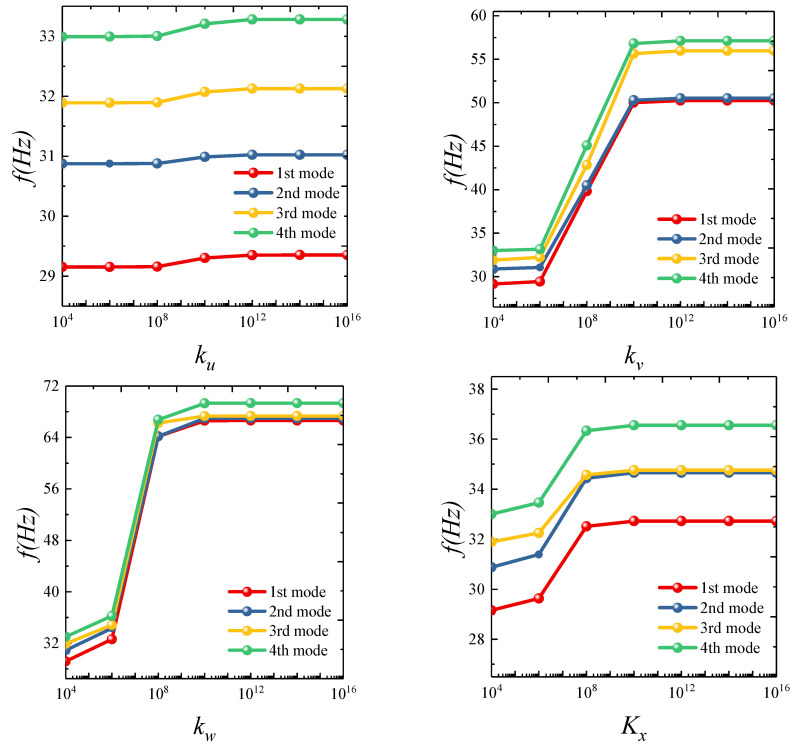

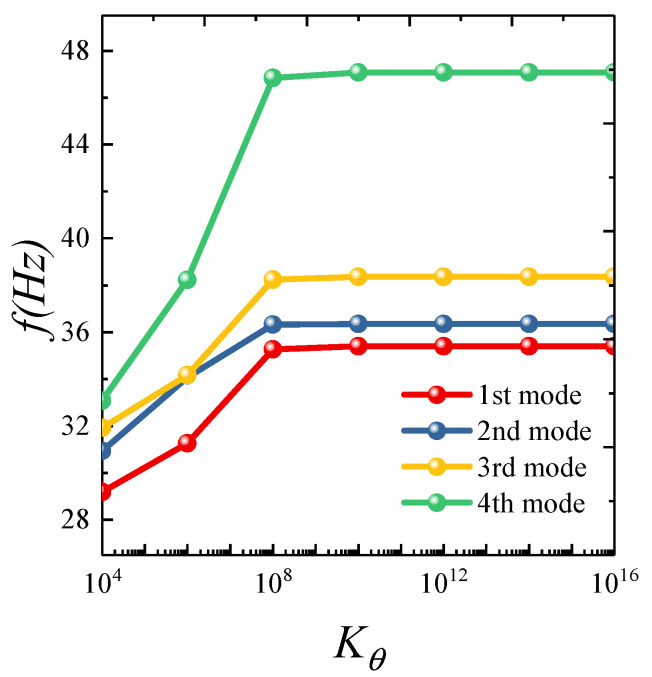
Convergence analysis of spring stiffness.

Subsequently, the influence of different boundary parameters on the natural frequencies of the elliptic cylinder was examined. To assess the role of various spring stiffness values in this study, only one spring stiffness was varied between 10^4^ and 10^16^, while the other four were kept at zero. Figure 3 presents a trend of the first four natural frequencies as each of the five spring stiffness values changes. It was observed that each spring stiffness exhibits a different sensitivity range. For instance, *k_v_* shows minimal frequency variation between 10^4^ and 10^6^, followed by an increase from 10^6^ to 10^10^, and stabilizes beyond 10^12^, indicating convergence. In contrast, *K_x_* has a sensitivity range from 10^6^ to 10^8^. Although this phenomenon supports the correctness of the previously set boundary condition parameters, three types of elastic constraint boundaries are further defined to clarify the effect of boundary spring stiffness on structural vibration characteristics: 

E1: $k_{v,0,1}=k_{w,0,1}{=10}^{8},\;k_{u,0,1}=K_{x,0,1}=K_{\theta ,0,1}=10^{15}$; 

E2: $k_{v,0,1}=k_{u,0,1}=K_{x,0,1}=K_{\theta ,0,1}=10^{15},k_{w,0,1}{=10}^{8}$; 

E3: $k_{v,0,1}=k_{u,0,1}=k_{w,0,1}{=10}^{15},$$K_{x,0,1}=K_{\theta ,0,1}=10^{8}$; 

E4: $k_{v,0,1}=k_{u,0,1}=k_{w,0,1}=K_{x,0,1}=K_{\theta ,0,1}=10^{8}$.

**Figure 4:**
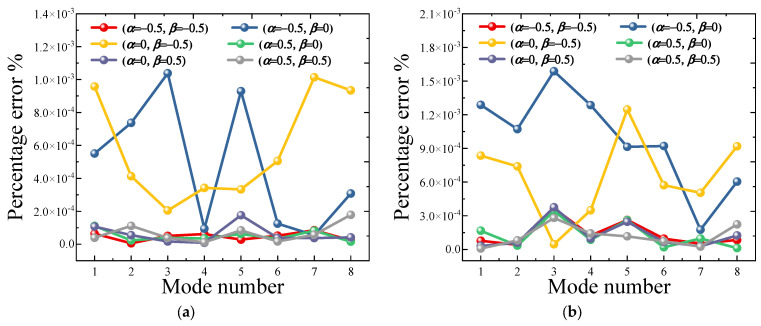

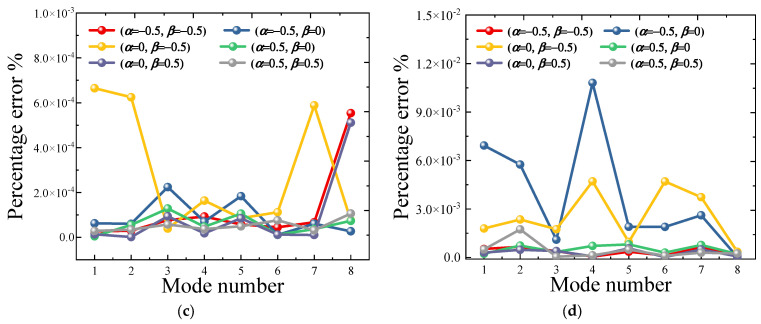
Convergence analysis of Jacobi coefficient (**a**) CC boundary; (**b**) SS boundary; (**c**) SD-SD boundary; (**d**) FC boundary.

Lastly, the influence of Jacobian parameters *α* and *β* on the vibration characteristics of the FG-CNTRC elliptic cylinder is further discussed. As we stated in the previous paper, because MVP and LSWRM are used, the boundary conditions need not be taken into account in the selection of displacement functions. This greatly reduces the inherent requirements for displacement functions. In this paper, we propose a unified Jacobian orthogonal polynomial with linear independence and regular differentiation to express the displacement admissible function. This means that when Jacobian parameters are different, different types of polynomial functions can be obtained. For example, several typical polynomials include the Chebyshev polynomial (*α* = *β* = −1/2), the second kind of Chebyshev polynomial (*α* = *β* = 1/2), and the Legendre polynomial (*α* = *β* = 0). Figure 4 shows the percentage error in the natural frequency obtained using different Jacobi parameters. Clearly, regardless of the choice of Jacobi parameters, the proposed method demonstrates high computational accuracy, with a maximum prediction error ($\left(\Omega_{\alpha ,\beta }-\Omega_{\alpha =0,\beta =0}\right)/\Omega_{\alpha =0,\beta =0}\times 100\%$) not exceeding $1.2\times 10^{-2}\%$. In subsequent computational analyses, the impact of varying parameters can be ignored. For consistency, we choose *α* = 0, *β* = 0 as the Jacobian parameters.

### 3.2. Vibration Analysis of FG-CNTRC Elliptic Cylinder

**Figure 5:**
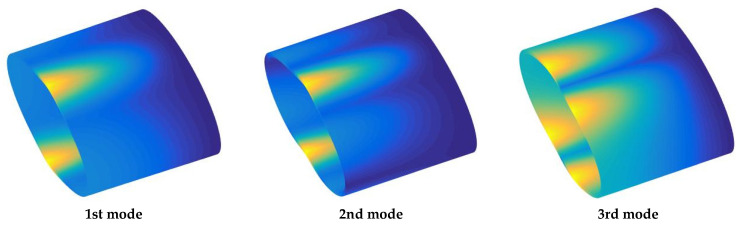

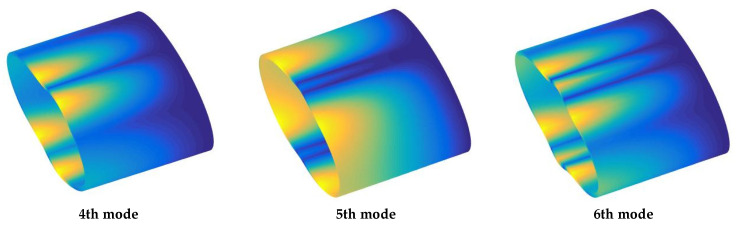
Mode shapes under *F-C* boundary conditions.

**Figure 6:**
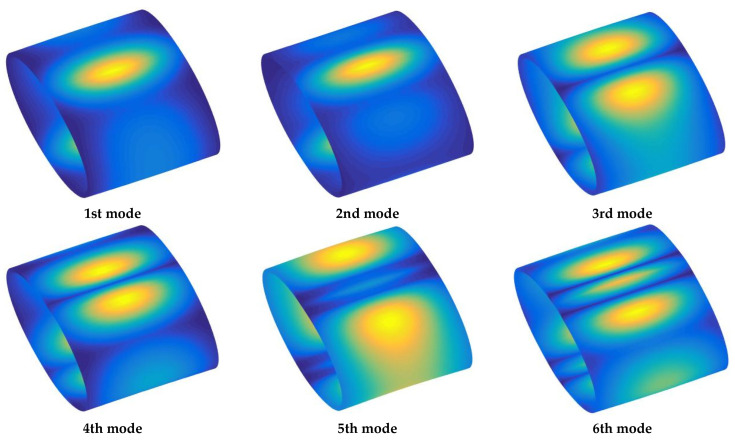
Mode shapes under *E*4-*E*4 boundary conditions.

This section begins with validation of the computational code’s accuracy. Table 1 presents a comparison between the results of this study and those reported in the existing literature. Ref. [43] used the modified variational method, DQM, and Ritz method, respectively. The geometric parameters used in the examples are: *a* = *b* = 1 m, *h* = 0.1 m, *L* = 2 m. The comparison demonstrates a high level of agreement, confirming the accuracy of the present method. It also shows that this method can effectively analyze the vibration characteristics of FG-CNTRC elliptical cylinders under arbitrary boundary conditions. Building on this, Table 2 and Table 3 further explore the variations in fundamental frequencies under several typical classical and elastic boundaries. The results clearly indicate that fundamental frequency parameters *Ω* are highly sensitive to boundary stiffness. As the boundary stiffness increases, the frequency rises significantly. Among the CNT distribution types studied, the frequency parameter is highest for the *X*-type distribution and lowest for the *O*-type distribution. Additionally, increasing CNT content also results in higher natural frequencies, suggesting that the inclusion of CNT medium contributes to greater dynamic stability of the structure. Finally, some mode shapes for *F*–*C* and *E*4–*E*4 FG-CNTRC elliptic cylinder are present in Figure 5 and Figure 6 by using the MATLAB SURF command. 

**Figure 7:**
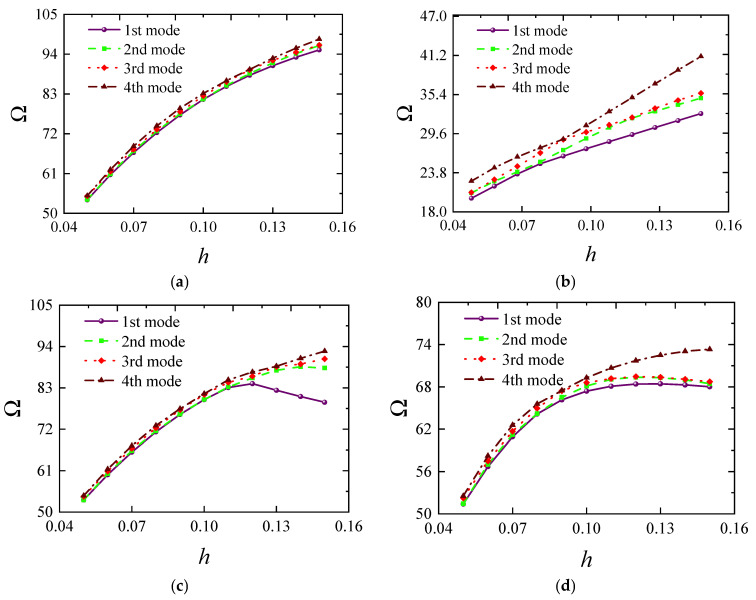
The influence of FG-CNTRC elliptical cylindrical shell thickness on frequencies Ω: (**a**) C-C boundary; (**b**) C-F boundary; (**c**) E1-E1; (**d**) E4-E4.

**Figure 8:**
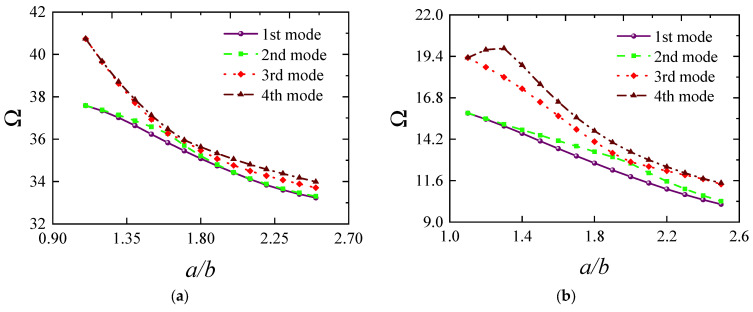

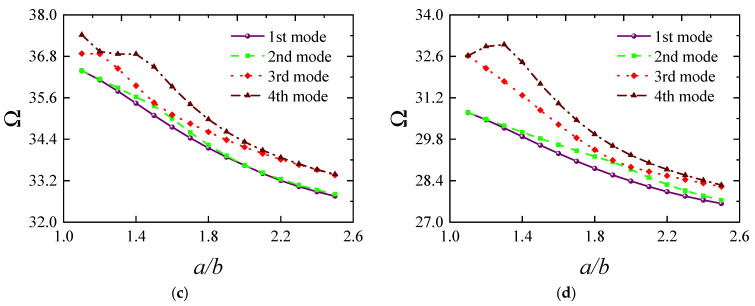
The effect of the radius ratio *a*/*b* of FG-CNTRC elliptical cylindrical shell on natural frequencies Ω: (**a**) C-C boundary; (**b**) C-F; (**c**) E1-E1; (**d**) E4-E4.

**Figure 9:**
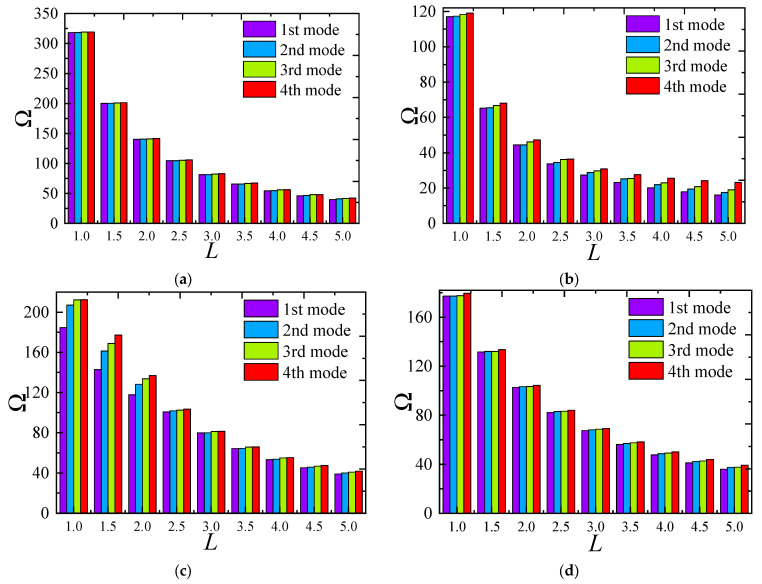
The effect of the length *L* of the FG-CNTRC elliptic cylindrical shell on natural frequencies Ω (**a**) C-C; (**b**) C-F; (**c**) E1-E1; (**d**) E4-E4.

**Figure 10:**
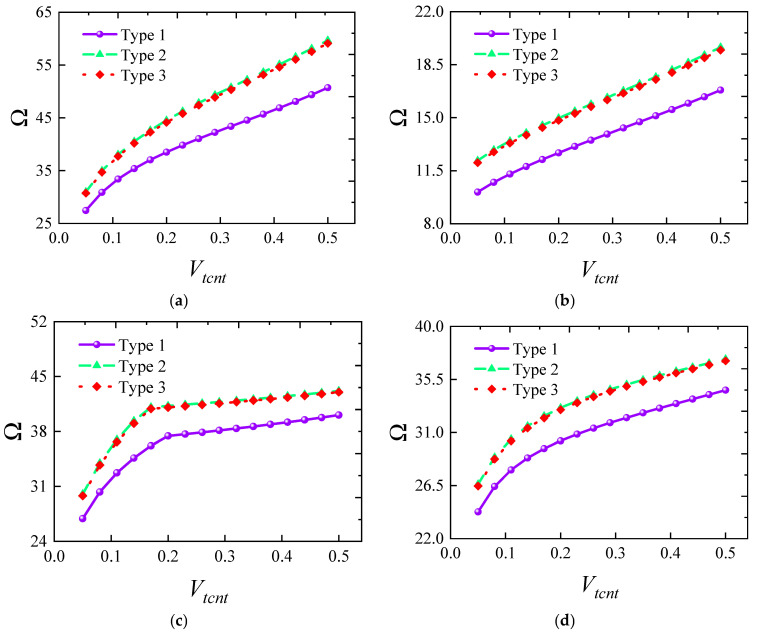
Variation in the fundamental frequency parameters Ω versus *V_tcnt_* for FG-CNTRC elliptic cylinder shells: (**a**) C-C; (**b**) C-F; (**c**) E1-E1; (**d**) E4-E4.

**Figure 11:**
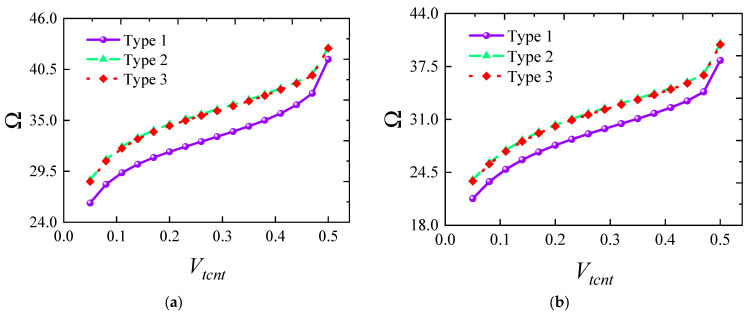

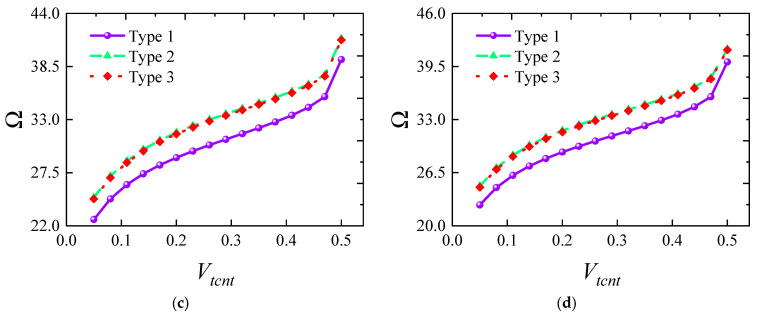
Variation in the fundamental frequency parameters Ω versus *V_tcnt_* for FG-CNTRC elliptic cylinder shells with different CNT types: (**a**) CNT-FGX; (**b**) CNT-FGO; (**c**) CNT-FGV; (**d**) CNT-FGΛ.

Figure 7 illustrates how the frequency of FG-CNTRC elliptical cylindrical shells varies with thickness under several typical boundary conditions. Generally, the fundamental frequency parameter *Ω* increases with thickness due to the substantial rise in structural stiffness. However, it is noteworthy that this trend does not always hold under elastic boundaries. For instance, under *E*1-*E*1 boundary conditions, the frequency starts to decrease when the thickness *h* exceeds 0.12. The remaining third-order frequencies also show a rapid increase followed by a gradual decrease, indicating that structural frequency is highly sensitive to elastic boundaries. Figure 8 shows the impact of the elliptical radius ratio on frequency, with results indicating a linear decrease in frequency as the radius ratio increases. A similar trend is confirmed in Figure 9, where the frequency parameter decreases as *L* increases. Figure 10 and Figure 11 respectively show the trend of fundamental frequency changes with CNT volume fraction *V_tcnt_* under various boundary conditions and distribution types. The two curves clearly indicate that an increase in *V_tcnt_* significantly raises the fundamental frequency, a phenomenon unaffected by boundary conditions or distribution types. However, different CNT distribution types lead to slight variations in the frequency trend.

## 4. Conclusions

This study introduces a unified analytical framework to analyze the vibration characteristics of FG-CNTRC elliptical cylindrical shells under various boundary conditions. Based on the findings, the following specific conclusions are drawn:

(1) The fundamental frequency of the elliptical cylindrical shells increases with a decrease in the length-to-radius ratio and an increase in the thickness-to-radius ratio, highlighting the critical role of geometry in structural stiffness.

(2) The incorporation of CNTs significantly enhances the natural frequencies of the shells. Among the studied CNT distribution types, the X-type distribution yields the highest frequency, whereas the O-type distribution results in the lowest, indicating the sensitivity of dynamic performance to material distribution.

(3) The Jacobi orthogonal polynomials employed in the displacement functions demonstrate excellent adaptability across all boundary conditions. Their generalization, encompassing Legendre and Chebyshev polynomials, ensures reliable prediction of dynamic responses.

(4) The proposed framework not only aligns closely with the existing literature but also extends its application to complex boundary and geometric configurations, providing valuable guidance for the design of advanced FG-CNTRC structures.

The practical significance of this work lies in its potential to advance the design and optimization of FG-CNTRC structures for critical applications in aerospace, maritime engineering, and construction industries, where high-performance and adaptable materials are increasingly essential. Future research will delve into the dynamic behavior of more intricate structural systems incorporating elliptical cylindrical shells. This includes exploring the frequency characteristics of composite structures formed by integrating elliptical shells with other components and addressing their practical applications in engineering.

## Figures and Tables

**Table 1:** Comparison of natural frequencies for FGM cylindrical shells under *F*-*C* boundary conditions (*h*/*R* = 0.1, *L*/*R* = 2, *R* = 1 m).

*p*	Method	Mode Number							
		1	2	3	4	5	6	7	8
0	Ref. [48]	152.93	152.93	220.06	220.06	253.78	253.78	383.55	383.56
	Ref. [49]	152.89	152.89	219.97	219.97	253.79	253.79	383.44	383.44
	Present	152.93	152.93	220.08	220.08	253.76	253.76	383.69	383.69
1	Ref. [48]	151.77	151.77	219.56	219.56	251.14	251.14	382.97	382.97
	Ref. [49]	151.52	151.52	219.19	219.19	250.81	250.81	382.35	382.35
	Present	151.77	151.77	219.59	219.59	251.12	251.12	383.10	383.10
5	Ref. [48]	148.97	148.97	218.87	218.88	244.40	244.40	382.46	382.47
	Ref. [43]	148.50	148.50	218.16	218.16	243.73	243.73	381.26	381.26
	Ref. [49]	148.53	148.53	218.21	218.21	243.76	243.76	381.33	381.33
	Present	148.97	148.97	218.90	218.90	244.38	244.38	382.60	382.60
20	Ref. [48]	146.46	146.46	215.90	215.90	239.84	239.84	377.34	377.34
	Ref. [43]	146.21	146.21	215.50	215.50	239.54	239.54	376.69	376.69
	Ref. [49]	146.24	146.24	215.55	215.55	239.57	239.57	376.76	376.76
	Present	146.47	146.47	215.92	215.92	239.83	239.83	377.47	377.49

**Table 2:** Frequency comparison of different CNT distribution types under various boundary conditions.

CNT-Type	*V_tcnt_*	Boundary Conditions							
		C-C	SD-SD	C-F	C-S	E1-E1	E2-E2	E3-E3	E4-E4
UD-CNT	0.12	34.11	22.30	11.45	27.97	33.41	30.51	32.65	28.20
	0.17	42.65	27.78	14.50	34.85	41.10	36.52	40.02	32.39
	0.28	48.39	31.95	16.03	39.96	41.51	39.45	44.50	34.34
FGX-CNT	0.12	37.06	24.86	12.21	30.88	36.21	32.39	35.17	29.68
	0.17	46.74	31.05	15.49	38.73	41.25	38.67	43.30	33.92
	0.28	53.09	36.40	17.58	44.70	41.96	41.71	48.22	35.85
FGO-CNT	0.12	29.00	19.06	10.58	23.62	28.41	26.98	28.20	25.28
	0.17	36.02	23.85	13.47	29.38	34.96	32.62	34.60	29.45
	0.28	41.68	26.96	14.79	33.80	40.43	36.19	39.29	32.00
FGV-CNT	0.12	31.38	20.71	11.19	25.98	30.63	28.76	30.33	26.67
	0.17	39.11	25.91	14.24	32.35	37.73	34.67	37.24	30.87
	0.28	45.09	29.56	15.69	37.37	42.02	38.16	42.10	33.24
FGΛ-CNT	0.12	31.13	20.08	10.71	25.85	30.61	28.49	30.10	26.59
	0.17	38.81	25.15	13.65	32.24	37.84	34.34	36.99	30.82
	0.28	45.00	29.07	15.27	37.50	41.74	38.03	42.09	33.28

**Table 3:** Frequency comparison of different CNT distribution types under classical-elastic boundaries.

CNT-Type	*V_tcnt_*	Boundary Conditions							
		CE1	CE2	CE3	CE4	SDE1	SDE2	SDE3	SDE4
UD-CNT	0.12	33.79	32.10	33.37	30.83	27.72	26.55	27.29	25.48
	0.17	42.17	39.07	41.32	36.72	34.41	32.40	33.66	30.30
	0.28	47.80	42.98	46.42	39.99	34.74	36.01	38.12	33.14
FGX-CNT	0.12	36.69	34.41	36.11	32.89	30.58	28.83	29.96	27.46
	0.17	46.16	41.91	45.00	39.15	35.09	35.15	37.08	32.50
	0.28	52.40	46.05	50.63	42.63	34.74	39.13	42.32	34.74
FGO-CNT	0.12	28.74	27.91	28.60	27.05	23.37	22.90	23.26	22.23
	0.17	35.58	34.13	35.30	32.47	28.96	28.17	28.77	26.85
	0.28	41.21	38.50	40.46	36.21	33.34	31.74	32.76	29.79
FGV-CNT	0.12	31.04	29.95	30.85	28.82	25.38	24.72	25.22	23.79
	0.17	38.56	36.59	38.15	34.47	31.46	30.36	31.19	28.51
	0.28	44.48	40.99	43.55	38.15	34.72	34.13	35.55	31.52
FGΛ-CNT	0.12	30.91	29.68	30.61	28.69	25.06	24.29	24.78	23.54
	0.17	38.43	36.28	37.88	34.34	31.13	29.84	30.66	28.26
	0.28	44.52	40.88	43.50	38.13	34.75	33.84	35.24	31.40

## Data Availability

The original contributions presented in this study are included in the article. Further inquiries can be directed to the corresponding author.
